# Robotic Release of Median Arcuate Ligament Syndrome: A Case Series

**DOI:** 10.7759/cureus.104287

**Published:** 2026-02-26

**Authors:** Khaled Y Omar, Natalie Ceballos, Shohab Virk, Christopher Garcia, Edilin Lopez, Michelle Gallas, Jorge Dorantes, Anthony Gonzalez

**Affiliations:** 1 General Surgery, Baptist Health South Florida, Miami, USA; 2 Colorectal Surgery, Greater Baltimore Medical Center, Baltimore, USA; 3 Thoracic Surgery, Lahey Clinic, Burlington, USA; 4 Center for Research, Baptist Health South Florida, Miami, USA

**Keywords:** celiac artery decompression, mals, median arcuate ligament syndrome, postprandial pain, robotic surgery, robotic surgical procedures, surgical case reports

## Abstract

Median arcuate ligament syndrome (MALS) is a rare condition caused by compression of the celiac artery by the median arcuate ligament (MAL), resulting in postprandial discomfort and abdominal pain. Surgical decompression remains the definitive treatment. We present a series of three patients with MALS, including a 22-year-old male, a 73-year-old male, and a 46-year-old female, managed with robotic median arcuate ligament release (MALR). All patients underwent diagnostic imaging with computed tomography angiography, demonstrating celiac artery stenosis with a characteristic hook-shaped appearance, as well as celiac artery duplex ultrasound revealing increased flow velocities with expiration. All procedures were performed using a robotic approach. After abdominal access and insufflation, the esophageal hiatus was exposed, the lesser omentum was opened, and the angle of His was dissected. The esophagus was encircled for retraction, allowing clear identification of the right and left crura and exposure of the aorta to the level of the celiac axis. Careful adhesiolysis enabled the complete release of the MAL along with surrounding neural and fibrous tissue. This case series highlights the importance of considering MALS in patients with chronic postprandial abdominal pain and demonstrates the feasibility of the robotic approach in offering enhanced visualization, precision, and dexterity to support the paucity of literature on the effectiveness of robotic MALR.

## Introduction

The median arcuate ligament (MAL) is a fibrous structure that is located superior to the celiac artery. MAL aids in connecting the two sides of the diaphragmatic crura. Usually, the MAL is at the level of the lower T12 vertebra at the aortic hiatus superiorly and anteriorly to the celiac axis. The celiac axis often displays variation in origin, ranging between T11 and L1. Median arcuate ligament syndrome (MALS) usually arises due to the descended insertion of the MAL or due to a higher origin of the celiac trunk. This anatomical variant results in dynamic stenosis, especially during expiration, potentially leading to ischemic symptoms. MALS is a rare disorder with an estimated incidence of 2 per 100,000 individuals [1]. It typically affects middle-aged females and is characterized by chronic postprandial abdominal pain, food aversion, weight loss, nausea, vomiting, and, occasionally, an abdominal bruit [2,3]. Many individuals are asymptomatic due to the abundant collateral circulation between the celiac and superior mesenteric arteries. However, the inability to compensate for this anatomic anomaly results in MALS [4].

Patients usually undergo extensive and diverse diagnostic studies that are not conclusive. Definitive diagnosis is achieved using imaging modalities such as computed tomography angiography (CTA), magnetic resonance imaging (MRI), and Doppler ultrasound. Published diagnostic criteria consist of either evident stenosis on CTA or a characteristic expiratory ‘flow increase’ of the celiac axis on duplex ultrasonography, defined by a peak systolic velocity >200 cm/seconds and an end-diastolic velocity >55 cm/second [5]. Management options include surgical decompression and an endovascular approach. Traditionally, median arcuate ligament release (MALR) is described extensively across the literature through open approaches. However, laparoscopic and robotic techniques have gained popularity due to reduced postoperative morbidity and superior surgical precision [4,6]. Robotic surgery offers advantages over a laparoscopic approach through improved visualization and dexterity [4,7]. However, due to the rarity of this disease, the operation is performed infrequently, and there is a significant lack of data on patients who have undergone robotic MALR to date [3,4].

We describe three cases of MALS managed via robotic decompression to contribute to the limited body of literature on the robotic minimally invasive approach [3,6].

## Case presentation

This is a retrospective single-center case series of three consecutive patients who presented between December 2017 and April 2019 and underwent robotic MALR for MALS. All patients presented with chronic postprandial epigastric pain and had undergone extensive prior diagnostic evaluations that were initially inconclusive. Despite heterogeneity in age, comorbidities, and prior surgical history, all cases shared hallmark clinical and radiographic features consistent with MALS.

The cohort included (1) a 22-year-old male with postprandial pain associated with nausea and vomiting and no significant past medical history; (2) a 73-year-old male with dysphagia, dermatomyositis, and severe protein-calorie malnutrition requiring percutaneous endoscopic gastrostomy tube feeding; and (3) a 46-year-old female with a history of gastric banding for morbid obesity and a concomitant hiatal hernia.

**Figure 1:**
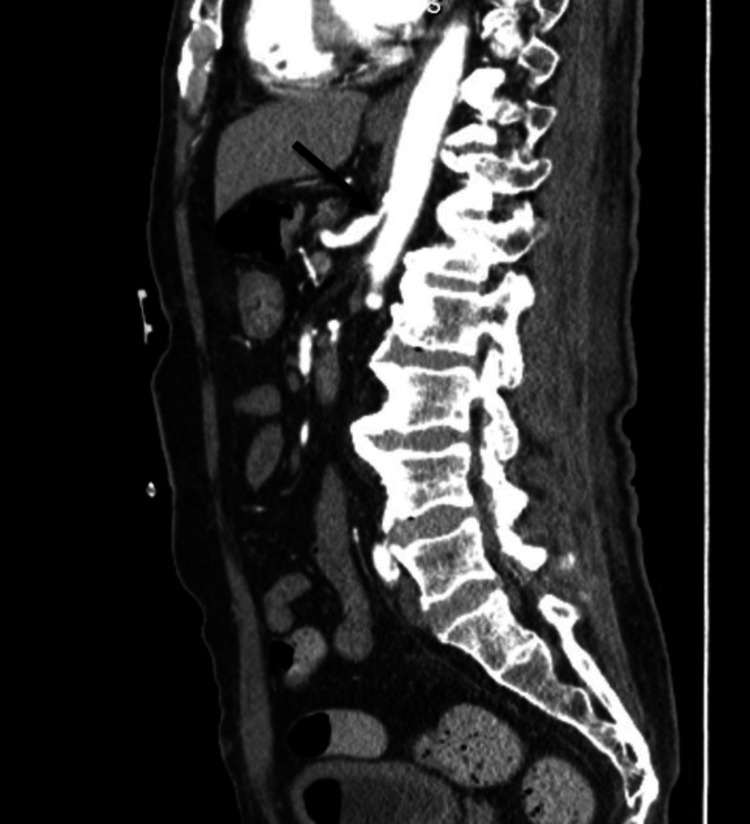
Computed tomography angiography sagittal plane showing celiac artery stenosis. The arrow is pointing to the celiac artery.

**Figure 2:**
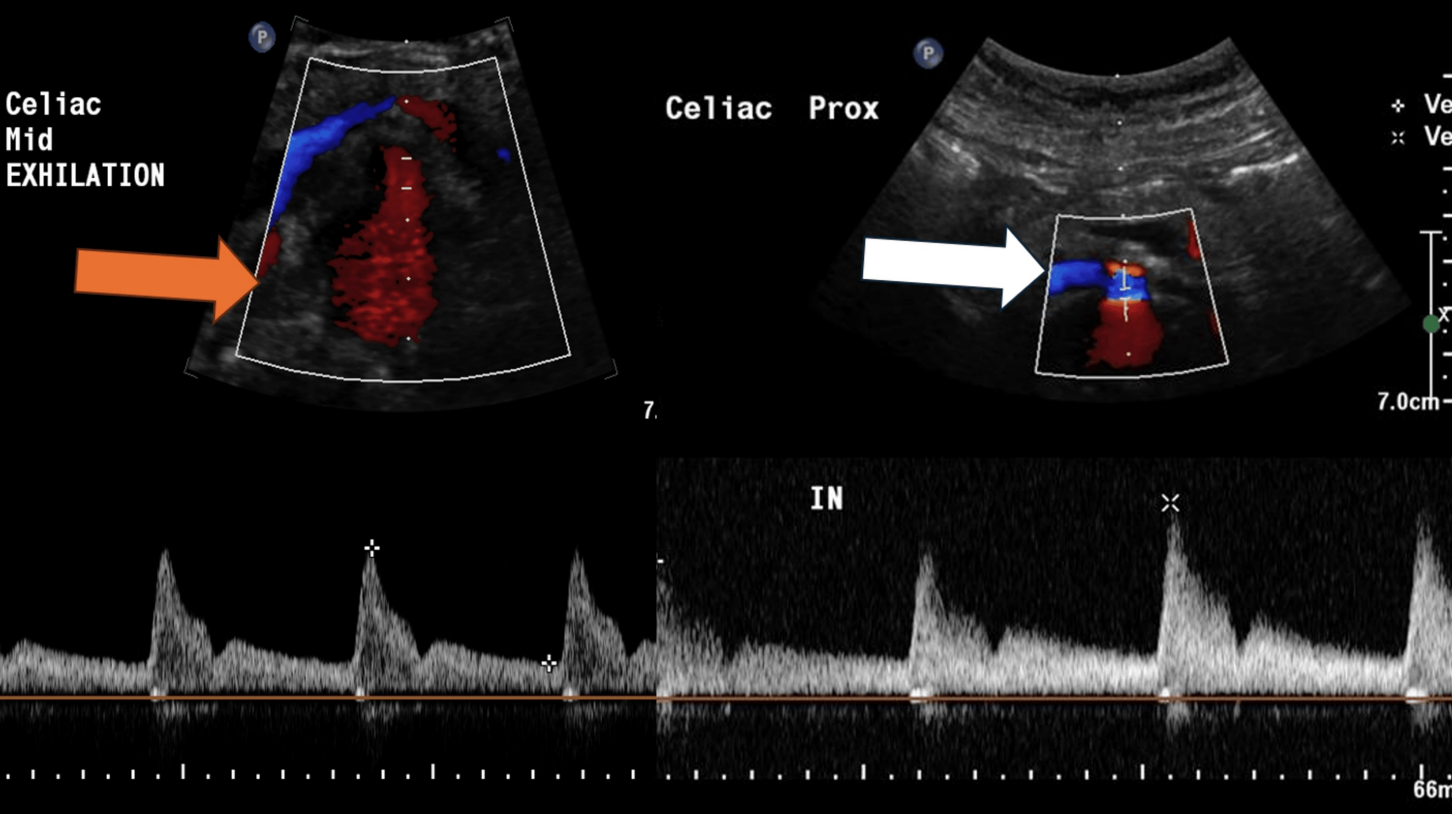
Duplex ultrasound of the celiac artery. The white arrow is pointing toward the celiac artery during inspiration. The orange arrow is pointing toward the celiac artery during expiration demonstrating increased flow during expiration.

All patients demonstrated consistent diagnostic findings on CTA, including celiac artery stenosis with a characteristic hooked configuration (Figure 1). Duplex ultrasound further supported the diagnosis, revealing elevated expiratory flow velocities with normalization during deep inspiration (Figure 2) (expiratory velocity up to 397 cm/second versus inspiratory velocity of approximately 150 cm/second), confirming dynamic extrinsic compression of the celiac axis.

**Figure 3:**
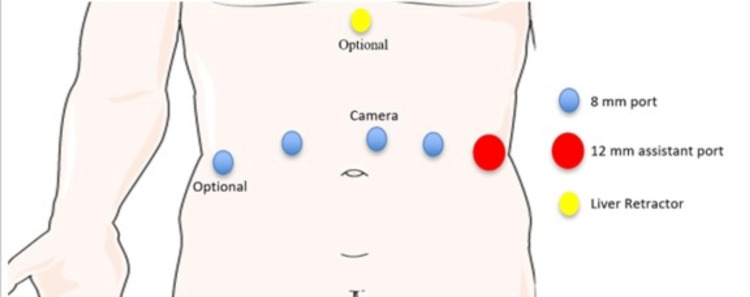
Port placement. Three 8-mm ports for the camera and two arms. One 12-mm assistant laparoscopic port. A liver retractor at the level of the falciform ligament. Image adapted from Servier Medical Art (https://smart.servier.com/), licensed under CC BY 4.0 (https://creativecommons.org/licenses/by/4.0/).

**Figure 4:**
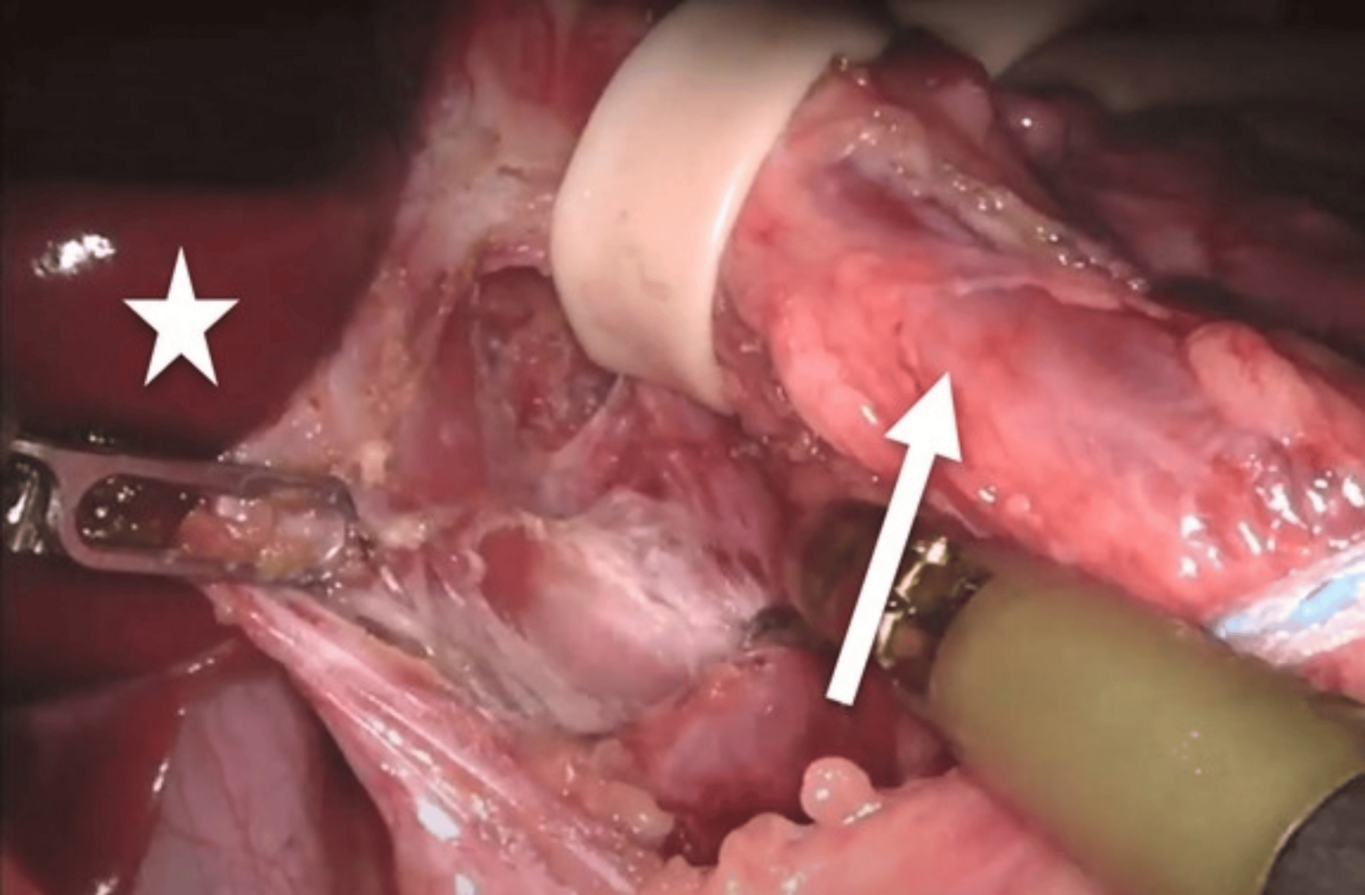
Separation of the right and left crus. The arrow is pointing toward the esophagus being retracted laterally by a Penrose drain. The star is marking the left lobe of the liver. Instruments aiding dissection included fenestrated bipolar forceps (left) and permanent cautery hook (right).

**Figure 5:**
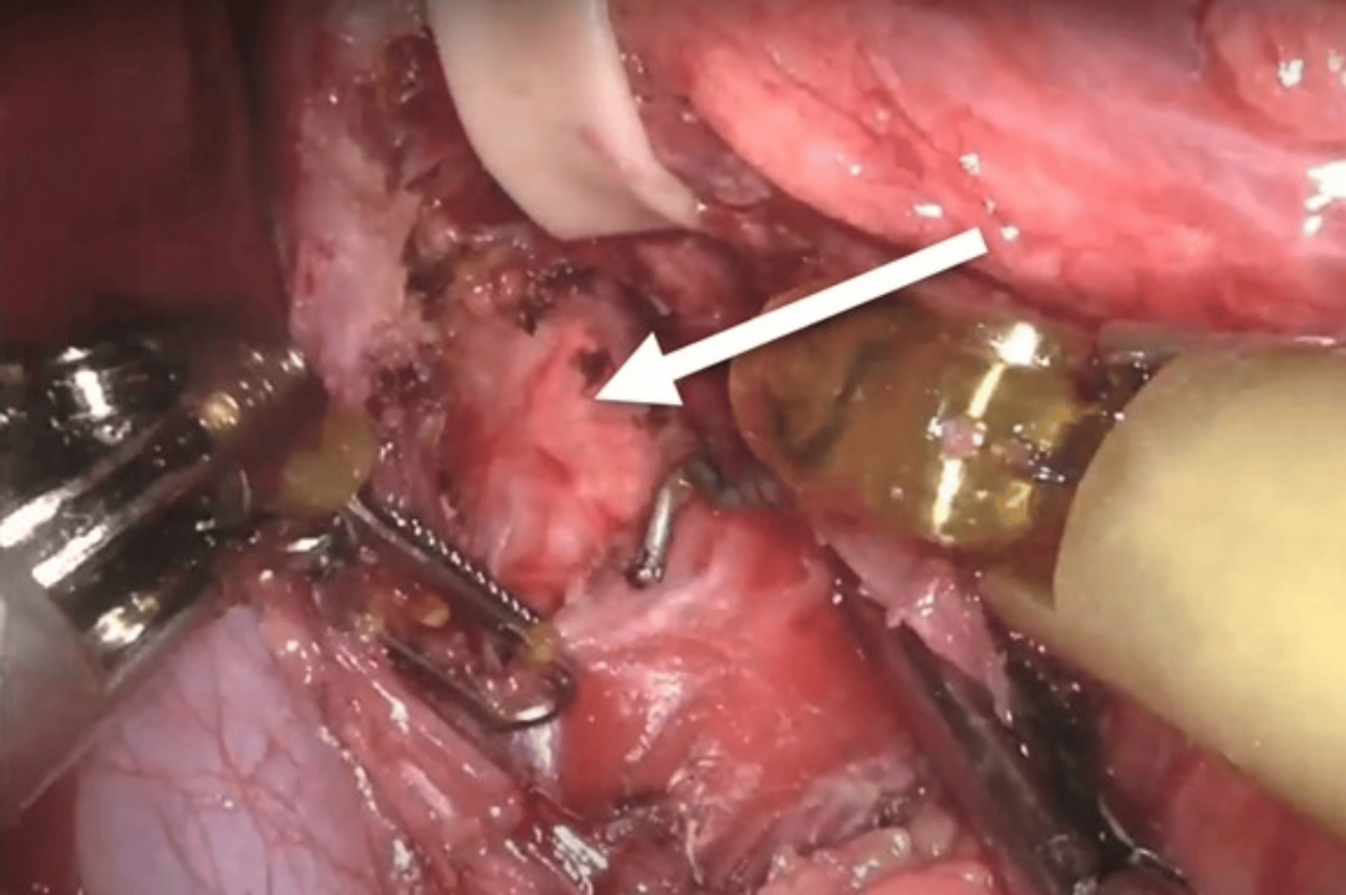
Caudal progression toward the celiac trunk. The arrow is pointing toward the aorta. Instruments aiding dissection included fenestrated bipolar lorceps (left) and permanent cautery hook (right).

**Figure 6:**
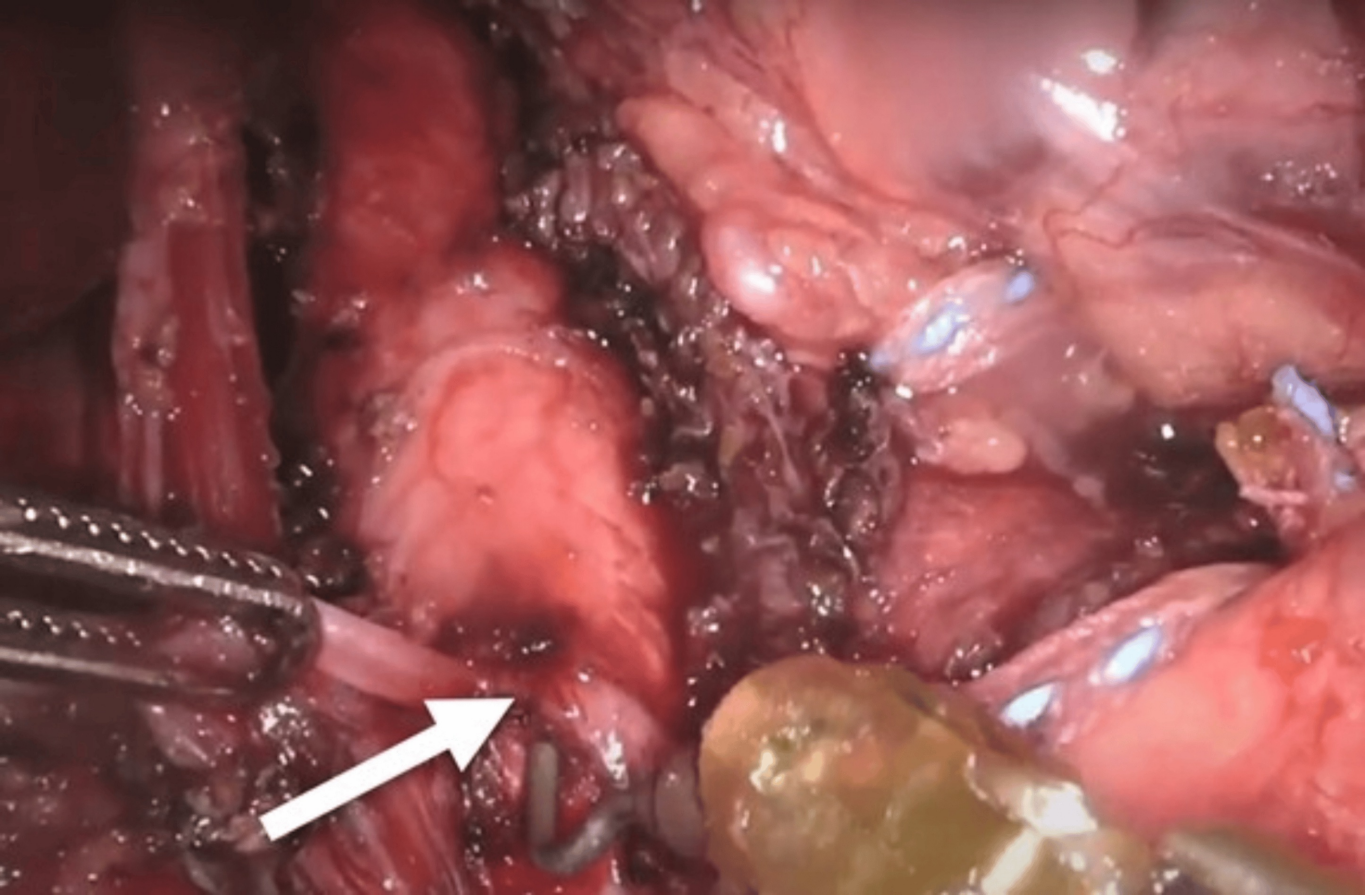
Celiac artery release. The arrow is pointing toward the celiac artery plexus release. Instruments aiding dissection included fenestrated bipolar forceps (left) and permanent cautery hook (right).

All procedures were performed robotically with the patient in the supine position. After standard preparation and insufflation, three 8-mm robotic trocars were placed under direct visualization. Two working ports were positioned along the right and left midclavicular lines, and a third port for the camera was placed slightly left of the midline, as depicted in the illustration in Figure 3. A Nathanson liver retractor was placed in the subxiphoid position. The lesser omentum was opened, and the angle of His was dissected. The right and left crura were carefully dissected using a vessel-sealing device. The esophagus was encircled with a Penrose drain for lateral retraction (Figure 4), allowing identification and separation of the crura and exposure of the aorta (Figure 5). Dissection proceeded distally to the celiac axis, where meticulous lysis of the MAL fibers, neural tissue, and surrounding adhesions was performed until complete decompression of the celiac artery was achieved (Figure 6). The aorta was also freed proximally to ensure adequate release. When present, the hiatal hernia was repaired using interrupted figure-of-eight 2-0 Ethibond sutures or a running 2-0 polypropylene barbed suture, while deliberately leaving the aortic hiatus widely patent.

At 30 days of follow-up, two of the three patients experienced significant improvement or resolution of postprandial abdominal pain. One patient continued to report persistent symptoms despite postoperative imaging confirming adequate celiac artery decompression. A comparative summary of patient characteristics, diagnostic findings, operative details, and outcomes is provided in Table 1 to synthesize key aspects of this case series.

**Table 1:** Summary of clinical characteristics, diagnostics, and outcomes. s/p: status post; PEG: percutaneous endoscopic gastrostomy; CTA: computed tomography angiography; MAL: median arcuate ligament

Characteristic	Case 1	Case 2	Case 3
Age/Sex	22/Male	73/Male	46/Female
Key symptoms	Postprandial epigastric pain, nausea, vomiting	Postprandial pain, dysphagia	Postprandial pain
Relevant comorbidities	None	Dermatomyositis, protein-calorie malnutrition	Morbid obesity (s/p gastric band), hiatal hernia
Prior interventions	None	PEG tube placement	Gastric banding
CTA findings	Hooked celiac artery stenosis		
Duplex ultrasound	↑ Expiratory / ↓ Inspiratory velocities		
Surgical approach	Robotic MAL release	Robotic MAL release	Robotic MAL release + hiatal hernia repair+ band removal
Postoperative duplex	Adequate decompression		
Symptom outcome	Improved	Persistent pain	Improved
Estimated blood loss (mL)	2	5	3
Length of stay (day)	1	10	1
Operative time (minutes)	110	119	46

## Discussion

MALS remains a challenging and controversial diagnosis, characterized by non-specific postprandial abdominal pain and frequently prolonged diagnostic delays. Surgical release of the MAL is currently the mainstay of treatment for symptomatic patients, with reported symptom improvement rates ranging from 60% to 80% across open, laparoscopic, and robotic approaches [8].

This case series highlights the feasibility, safety, and potential clinical benefits of robotic-assisted MALR. In our series of three patients with heterogeneous clinical backgrounds, all procedures were completed robotically without conversion, and no intraoperative complications occurred. Two of the three patients experienced meaningful postoperative symptom improvement, findings that align with previously published outcomes for minimally invasive MALR [8].

Comparison with existing literature

Historically, open MALR was considered the gold standard, offering direct tactile feedback and reliable exposure of the celiac axis. Early series reported symptom improvement in approximately 70-80% of patients, but at the expense of increased postoperative pain, longer hospital stays, and higher morbidity compared with minimally invasive techniques [9]. As laparoscopic surgery gained adoption, multiple studies demonstrated comparable symptom relief with reduced length of stay and faster recovery [10]. However, laparoscopic MALR remains technically demanding due to limited instrument articulation, two-dimensional visualization, and the need for meticulous dissection around the aorta and celiac plexus.

Robotic-assisted MALR has emerged as an evolution of minimally invasive management, aiming to overcome these technical limitations. Several small case series and retrospective studies have demonstrated encouraging results. Gerull et al. reported symptom improvement in approximately 80% of patients undergoing robotic MALR, with low complication rates and no need for conversion to open surgery [11]. Similarly, authors have highlighted the advantages of robotic visualization and wristed instrumentation in achieving precise circumferential decompression of the celiac artery and associated neural tissue [12].

Our findings are consistent with these reports. The robotic platform provided excellent exposure of the supraceliac aorta and celiac axis, facilitating careful lysis of the MAL fibers and perivascular neural tissue. The enhanced dexterity and tremor filtration allowed controlled dissection in a confined and anatomically variable region, which is particularly relevant in patients with prior foregut surgery or complex anatomy, as demonstrated in our third case.

Clinical outcomes and symptom persistence

Despite successful anatomic decompression on postoperative imaging, one patient in our series continued to experience abdominal pain. Persistent symptoms following technically successful MALR have been described in up to 20-40% of patients in prior studies [8]. Proposed explanations include irreversible neurogenic pain, coexisting functional gastrointestinal disorders, psychiatric comorbidities, another unknown cause of the abdominal pain, or chronic ischemic changes that do not fully reverse after decompression. This underscores the importance of careful patient selection and comprehensive preoperative evaluation.

Several authors have emphasized that radiographic compression alone does not reliably predict postoperative symptom relief. Duplex ultrasound findings, respiratory variation, symptom reproduction, and exclusion of alternative diagnoses remain critical components of preoperative assessment [13]. Our experience reinforces the multifactorial nature of postoperative outcomes in MALS and highlights the need for realistic patient counseling.

Advantages of the robotic platform

While no randomized trials directly compare robotic and laparoscopic MALR, available evidence suggests comparable symptom relief with potential ergonomic and technical advantages favoring the robotic approach [14]. These advantages include three-dimensional magnified visualization, improved surgeon ergonomics, wristed instrumentation for fine dissection, and enhanced precision when working adjacent to major vascular structures. In our series, these features contributed to the safe completion of all cases without complications or conversion, even in patients with significant comorbidities.

Study limitations

Continued reporting of institutional experiences and longer-term follow-up are needed to better define patient selection criteria and further clarify the role of robotic surgery in the management of MALS. This study is limited by its small sample size and retrospective design. The absence of a comparison group undergoing open or laparoscopic release limits definitive conclusions regarding the superiority of the robotic approach. Additionally, symptom improvement was primarily assessed through subjective patient reporting rather than standardized quality-of-life instruments. Follow-up duration was limited, precluding assessment of the long-term durability of symptom relief. Future research should focus on multicenter prospective studies comparing robotic, laparoscopic, and open approaches, with standardized outcome measures and longer follow-up. Refinement of preoperative selection criteria, including functional imaging, pain characterization, and psychosocial assessment, may improve patient outcomes and reduce persistent postoperative symptoms. Incorporating validated quality-of-life metrics and cost-effectiveness analyses will further clarify the role of robotic surgery in the evolving treatment algorithm for MALS.

## Conclusions

This retrospective single-center series of three consecutive patients demonstrates that robotic MALR is a safe and feasible approach for the treatment of MALS. In this cohort, the robotic platform facilitated precise exposure and complete release of the MAL and surrounding fibrotic tissue, with favorable short-term postoperative outcomes at 30-day follow-up. These cases support the role of a robotic approach as an effective minimally invasive option in appropriately selected patients with imaging-confirmed MALS.
